# Miscellaneous Notices

**Published:** 1851-04-01

**Authors:** 


### Miscellaneous Notices.

*God in Disease.* By J. F. Duncan, M.D., &c. London, 1851.

This is an excellent idea, executed on the plan of the "Bridgewater Treatises." The learned and Christian author enters fully into the consideration of his subject, and has written a work both entertaining and highly instructive. He observes that most persons are in the habit of admitting that the visitation of sickness is the result of the direct appointment of God; but scarcely any one appears to think that such an admission implies the existence of features stamped upon the dispensation, similar to what are to be found on other parts of the divine proceedings, and that are eminently deserving of being studied carefully. Dr. Duncan's work is intended to direct attention to the subject, and to unfold, by an analysis of the phenomena of disease, the evidence of design, contrivance, and beneficence, that lie scattered in profusion over every page of the volume of natural history. In illustrating his subject, the author has availed himself of all the light that the progress of pathological science in recent times has placed at his command. We can strongly recommend this little volume to the notice of our readers. The work forms an admirable present for students, and even the more advanced members of our profession cannot peruse it without much pleasure and instruction.

*The Bath Waters: their Uses and Effects in the Cure and Relief of Chronic Diseases.* By James Tunstall, M.D. London. J. Churchill.

Dr. Tunstall is a physician of eminence at Bath. He has devoted considerable attention to the administration of the Bath waters in the cure of disease. We believe he is the first physician of late years who has taken this subject up. The work bears evidently the impress of being written by a well-educated, intelligent, and practical man. The chapter on "Brain Fag," is full of valuable suggestions. As Bath is so often resorted to by invalids, it will be a great comfort for them to know that they will have an opportunity of availing themselves of the celebrated waters of that beautiful city under the instructions of a physician fully acquainted with their many virtues. We believe that it is too commonly the practice of invalids, in visiting our celebrated spas, to exercise their own judgment in the use of the waters, and, consequently, in many cases, they return very little benefited from their administration. No invalid should seriously think of going through a course of mineral waters without consulting a physician who has well studied their effects and varied influence on different constitutions, and different diseases. At Bath, the invalid will have no difficulty in this matter. Dr. Tunstall is a scholar, a gentleman, and a sound practical physician, and is conversant with the use of the mineral waters. We can strongly recommend his work to the perusal of the profession and the public.

*Facts and Observations in Medicine and Surgery.* By JOHN GRANTHAM, F.R.C.S. London.

THIS work contains some valuable observations on points connected with cerebral pathology and therapeutics, and it is on this account that we are induced to bring it under the notice of our readers. The chapters we refer to are—1. On the premonitory symptoms of insanity; 2. The management of lunatics; 3. On epilepsy; 4. Cerebral affections from deficiency in the cranium; 5. Effects of deficient ossification of the cranium; 6. On the phosphatic deposits in the urine of children. All these chapters contain matter of deep interest to the psychological physician. Mr. Grantham has no disposition to soar aloft either in metaphysical or psychological science. He writes like a practical man who has not been satisfied with *seeing* without *observing*. Our crowded pages deprive us of the pleasure of extracting several passages which we had marked in the book. We have been much pleased with the work, and can strongly recommend it to our readers.

*On Medicines: their Uses and Mode of Administration.* By J. M. NELIGAN, M.D., M.R.I.A. Third Edition. Dublin. Fannin. 1851.

THIS is one of the most valuable works that has been published of late years. It is an admirable volume of reference, and ought to be found in the library of every practising physician. It contains a vast body of valuable facts connected with the art of administering medicines, most lucidly arranged, written by a physician who has evidently thought for himself. Let our readers procure the work, and judge for themselves.

*God and Man.* By ROBERT MONTGOMERY, M.A., Oxon. London. 1850.

THIS is strictly a theological work, and, as such, does not come legitimately within the scope of this Journal. It is an able production, and is written in glowing and eloquent language. Mr. Montgomery has a vigorous understanding, and he never takes up his pen without writing what is worth reading and remembering.

*The Illustrated Book of Songs.* W. S. Orr & Co. London.

THIS is an elegantly got-up work. The illustrations are of the highest order. We have seldom seen a volume of its size containing so many really beautiful engravings. The songs, the editor informs us, are principally translations from the German. They are poetical, and many of them touch the heart in its tenderest chords. It affords us much pleasure to speak in terms of warm commendation of this little book.

*On Excision of the Enlarged Tonsils in Cases of Deafness.* By W. HARVEY, Surgeon. London. 1850.

MR. HARVEY has devoted much attention to the study and treatment of ear affections, and everything that falls from his pen is entitled to our best attention. There are many points of practical interest in the work, which will commend it to those interested in this branch of pathology.

*A Letter to Lord Campbell on the clause respecting Chloroform, in the proposed Prevention of Offences Bill.* By J. SNOW, M.D. 1851.

A TIMELY and well-written pamphlet. We are afraid, however, it is a hopeless task for medical men to attempt to convince the learned gentlemen of the long robe of the error of their ways. There is, unfortunately, but little disposition on the part of the bench or the bar to listen to medical authority. We wish it were otherwise. Lord Campbell is an able, a liberal, and a discriminating judge, and we hope he will "read, mark, learn, and inwardly digest" Dr. Snow's pertinent observations on the impossibility of chloroform becoming a common mode of creating insensibility with the view of facilitating the commission of offences against the person.

